# Meso/Microporous Carbons from Conjugated Hyper-Crosslinked Polymers Based on Tetraphenylethene for High-Performance CO_2_ Capture and Supercapacitor

**DOI:** 10.3390/molecules26030738

**Published:** 2021-01-31

**Authors:** Mohamed Gamal Mohamed, Mahmoud M. M. Ahmed, Wei-Ting Du, Shiao-Wei Kuo

**Affiliations:** 1 Department of Materials and Optoelectronic Science, Center of Crystal Research, National Sun Yat-sen University, Kaohsiung 804, Taiwan; mgamal.eldin34@gmail.com (M.G.M.); mahmoud.ahmed@mail.ntust.edu.tw (M.M.M.A.); justguita@gmail.com (W.-T.D.); 2 Chemistry Department, Faculty of Science, Assiut University, Assiut 71516, Egypt; 3 Chemistry Department, Chung Yuan Christian University, Taoyuan 320, Taiwan; 4 Department of Medicinal and Applied Chemistry, Kaohsiung Medical University, Kaohsiung 807, Taiwan

**Keywords:** Friedel–Crafts reaction, porous polymers, triazine covalent framework, CO_2_ capture, supercapacitor

## Abstract

In this study, we successfully synthesized two types of meso/microporous carbon materials through the carbonization and potassium hydroxide (KOH) activation for two different kinds of hyper-crosslinked polymers of TPE-CPOP1 and TPE-CPOP2, which were synthesized by using Friedel–Crafts reaction of tetraphenylethene (TPE) monomer with or without cyanuric chloride in the presence of AlCl_3_ as a catalyst. The resultant porous carbon materials exhibited the high specific area (up to 1100 m^2^ g^−1^), total pore volume, good thermal stability, and amorphous character based on thermogravimetric (TGA), N_2_ adsoprtion/desorption, and powder X-ray diffraction (PXRD) analyses. The as-prepared TPE-CPOP1 after thermal treatment at 800 °C (TPE-CPOP1-800) displayed excellent CO_2_ uptake performance (1.74 mmol g^−1^ at 298 K and 3.19 mmol g^−1^ at 273 K). Furthermore, this material possesses a high specific capacitance of 453 F g^−1^ at 5 mV s^−1^ comparable to others porous carbon materials with excellent columbic efficiencies for 10,000 cycle at 20 A g^−1^.

## 1. Introduction

The developing new storage technologies and other energy resources with high specific power and energy storage capability such as supercapacitors and batteries have become an important topic in both the academic and industry areas [1]. The replacing of fossil fuels by designing devices like supercapacitors and batteries can reduce and prevent global warming, inadequate environment, and polluted atmosphere [2,3,4,5,6,7]. Ultra-capacitors or electric double layer capacitors (also called supercapacitors) are energy storage devices that possess long cycle life, low maintenance, low internal resistance, light weight, delivering high energy and high efficiency, high power, flexible packaging, light weight and maintenance, and a wide range of temperature compared with other energy devices such as batteries [8,9,10,11,12,13,14,15]. Based on the supercapacitors mechanism for energy storage, supercapacitors can be divided into electrical double layer capacitor (EDLC), hybrid capacitor, and finally pseudocapacitor [16,17,18,19,20,21,22,23,24,25,26,27]. Hyper-crosslinked polymers (HCPs) [28,29,30,31,32,33], covalent organic frameworks (COF) [34,35,36,37,38,39,40], conjugated microporous polymers (CMPs) [41,42,43,44,45], and polymers of intrinsic microporosity (PIMs) are considered as types of microporous organic polymers (MOPs) [46,47,48,49]. As mentioned above, HCPs are considered as a kind of porous material that can be easily synthesized through the Friedel–Crafts alkylation reaction of rigid aromatic monomers with an external crosslinker such as formaldehyde dimethyl acetal, 1,3,5-trichlorotriazine, and 1,4-bis(chloromethyl)benzene [50,51,52]. Compared with other microporous material such as activated carbon, HCPs can be synthesized by low-cost monomers, catalysts, and suitable reaction conditions [53,54]. In addition, HCPs materials feature easy functionalization, good chemical and thermal properties, microporous nature, and large surface areas [50,51,52,53,54]. Nowadays, the preparation of porous carbon materials has attracted much attention due to their interesting properties such as excellent electrical conductivity, high surface areas and high pore volumes, and good chemical, thermal, and mechanical stabilities [55,56,57,58,59]. Therefore, porous carbonaceous materials have been applied in many real-life applications, for example catalysis, gas separation, gas capture, energy storage in supercapacitors and batteries, fuel cells, water treatment and purification, and electromagnetic interface shielding [60,61,62,63,64]. As reported, the preparation of porous carbonaceous materials with high surface area and excellent porosity nature can be achieved by chemical activation for many polymers’ precursors, for example conjugated microporous polymers (CMPs), metal-organic frameworks (MOFs), HCPs, and porous aromatic frameworks (PAFs) [62,63,64]. Herein, we successfully prepared porous carbon materials derived from KOH activation at 800 °C of TPE-CPOP1 and TPE-CPOP2 as hypercross-linked polymers, which were prepared by the Friedel–Crafts polymerization of tetraphenylethene with or without cyanuric chloride as an external crosslinker in the presence of AlCl_3_ as a catalyst. The TGA, XPS, Raman, BET, and PXRD measurements were used to understand and determine their thermal stability, chemical compositions, surface areas, and crystallinity properties. Furthermore, the electrochemical and CO_2_ uptake analyses for TPE-CPOP1 and TPE-CPOP2 after thermal treatment at 800 °C were done to investigate their potential application in energy storage and gas capture.

## 2. Results

### 2.1. Synthesis and Character of TPE-CPOP1 and TPE-CPOP2

**Scheme 1:**
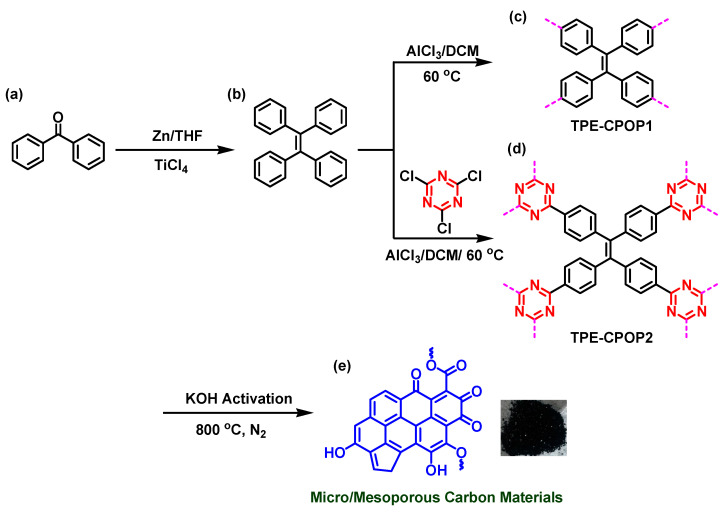
Preparation of (**b**) tetraphenylethene (TPE), (**c**) TPE-CPOP1 (conjugated porous organic polymer), (**d**) TPE-CPOP2, and (**e**) microporous carbon materials from benzophenone (**a**).

Scheme 1 shows the synthetic route for the preparation of hyper-crosslinked polymers and microporous carbon materials from TPE as a building monomer. Firstly, the TPE monomer was synthesized through the reaction of benzophenone with zinc and titanium tetrachloride (TiCl_4_) in the presence of THF at 80 °C to give TPE as a white solid. Secondly, TPE-CPOP1 and TPE-CPOP2 were prepared through a simple Friedel–Crafts reaction of TPE monomer with or without cyanuric chloride in the presence of anhydrous 1, 2-dichloroethane as solvent and AlCl_3_ as a catalyst (Scheme 1a–d). Finally, TPE-CPOP1-800 and TPE-CPOP2-800 were prepared through the carbonization and KOH activation process for their corresponding polymers as presented in Scheme 1e.

**Figure 1:**
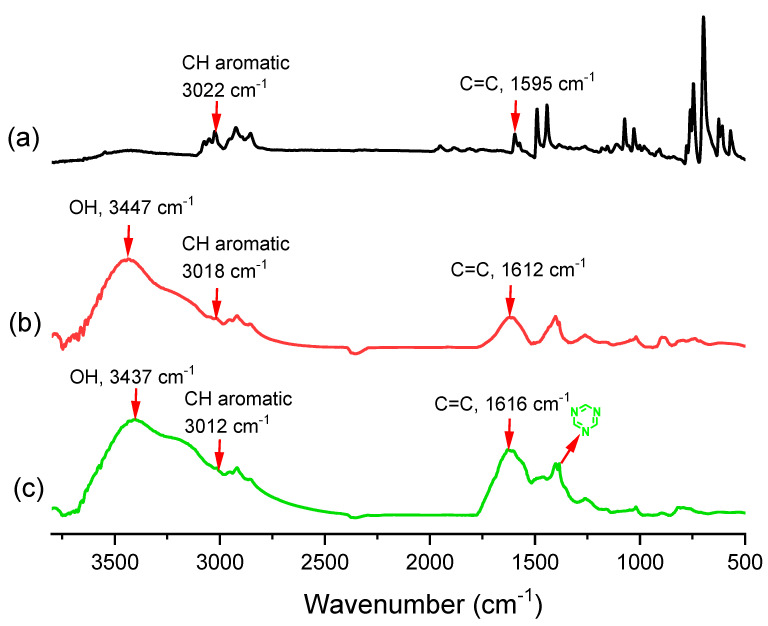
FTIR spectra of (**a**) TPE, (**b**) TPE-CPOP1, and (**c**) TPE-CPOP2.

The spectral analyses of TPE monomer were in agreement with our reported results (Figures S1 and S2). The chemical molecular structure of TPE-CPOP1 and TPE-CPOP2 was carefully confirmed by using FTIR and the solid state ^13^C NMR spectra analyses. Figure 1 presents the FTIR profile of TPE monomer, TPE-CPOP1 and TPE-CPOP2, respectively, recorded at room temperature. The characteristics absorption bands appeared at 3022 and 1599 cm^−1^ corresponding to the stretching C–H aromatic groups and C=C bonds as displayed in FTIR spectrum of TPE (Figure 1a). Meanwhile, the FTIR spectra (Figure 1b,c) of both TPE-CPOP1 and TPE-CPOP2 showed absorption bands at ca. 3447, 3022 and 1595 cm^−1^, which are attributed to the presence of a hydroxyl group from adsorbed water by these porous materials, the stretching C–H aromatic groups and C=C bonds, respectively.

**Figure 2:**
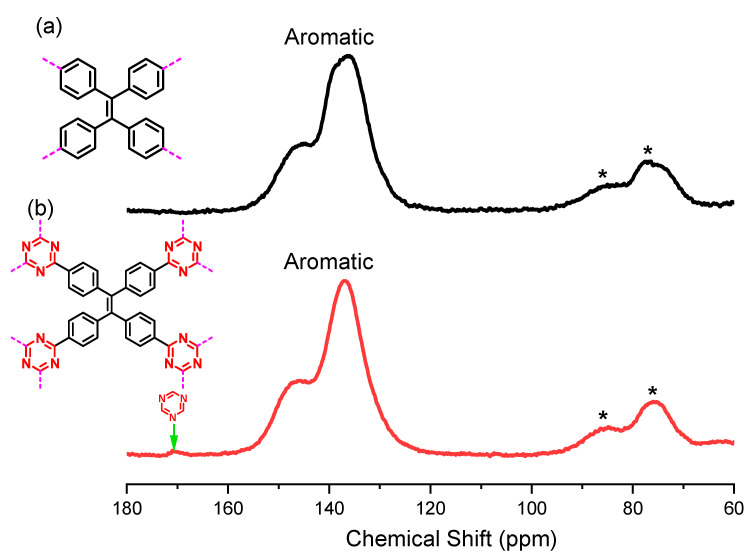
Solid state ^13^CNMR spectra (**a**) TPE-CPOP1 and (**b**) TPE-CPOP2. where * is the side band of solid-state NMR.

The chemical structure of TPE-CPOP1 and TPE-CPOP2 was further analyzed by solid-state ^13^C NMR measurements (Figure 2). The appearance peaks in the range 145.80–136.51 ppm in TPE-CPOP1 (Figure 2a) and 147–137 ppm in TPE-CPOP2 (Figure 2b), respectively; corresponded to the aromatic carbon nuclei. In addition, the peaks about 171 ppm in the TPE-CPOP2 could be assigned to the C=N in triazine units (Figure 2b).

**Figure 3:**
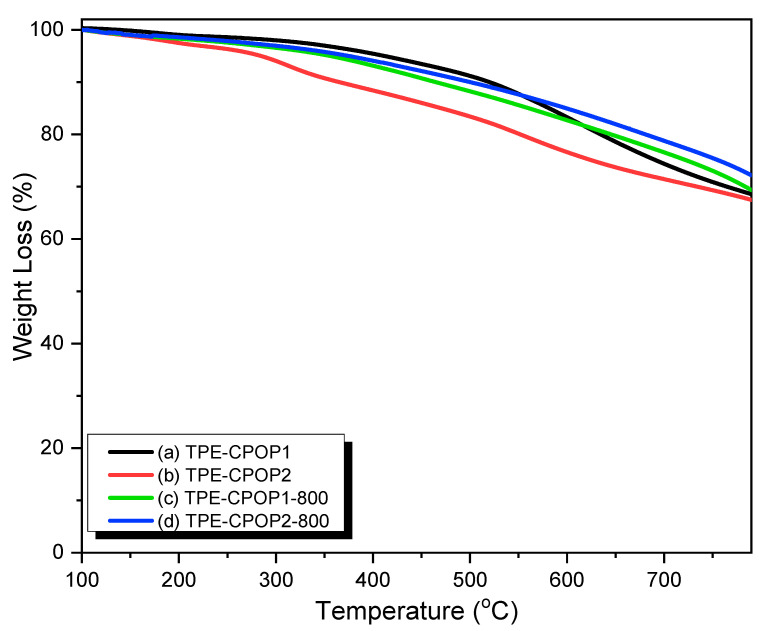
TGA diagram of (**a**) TPE-CPOP1 and (**b**) TPE-CPOP2, (**c**) TPE-CPOP1-800, and (**d**) TPE-CPOP2-800.

Based on FTIR and solid-state NMR analyses showed the successful incorporation of the triazine ring into the TPE-CPOP2 framework. The thermal stability of porous materials is important for the real-life application. Thus, the thermal stability of TPE-CPOP1 and TPE-CPOP2, TPE-CPOP1-800, and TPE-CPOP2-800 were determined by thermogravimetric analysis (TGA) (Figure 3, Table 1). The values of degradation temperatures when the weight loss of the sample reached 5% (T_d5_), 10% (T_d10_), and char yield for TPE-CPOP1 and TPE-CPOP2 were 412, 519 °C, and 69% and 284, 365 °C, and 67%, respectively. Meanwhile, after carbonization and KOH activation at 800 °C for 8 h, the degradation temperatures (T_d5_ and T_d10_) and char yield were 365, 464 °C, and 70%, respectively, for TPE-CPOP1-800 and 375, 500, and 72%, respectively, for TPE-CPOP2-800. According to TGA results, our materials featured good thermal stability comparable with other porous materials. As displayed in Figure S4, the XPS survey spectra showed signals at 284 eV and 530 eV representing the carbon atoms of the aromatic rings and oxygen atoms in both TPE-CPOP1 and TPE-CPOP2. In addition, the XPS profile of TPE-CPOP2 displayed signal at 400 eV representing the nitrogen atoms in the triazine units. 

**Figure 4:**
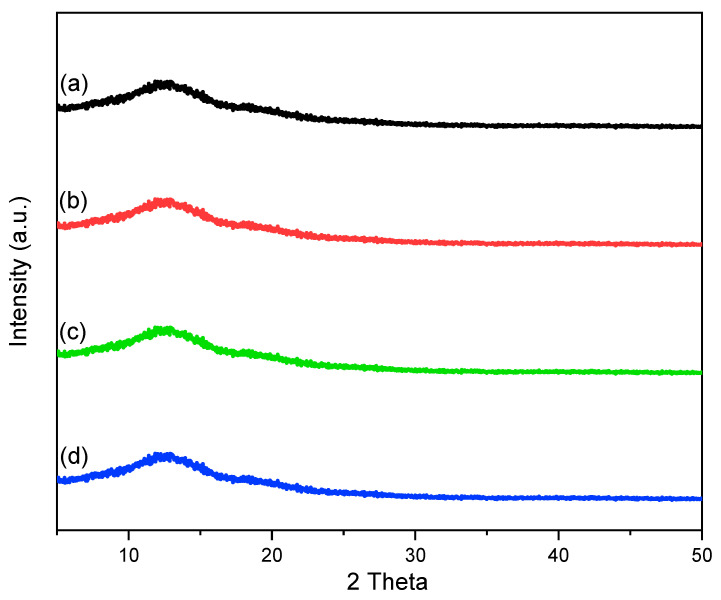
PXRD pattern of (**a**) TPE-CPOP1 and (**b**) TPE-CPOP2, (**c**) TPE-CPOP1-800, and (**d**) TPE-CPOP2-800.

All the as-synthesized materials in this study appeared a broad peak ca. 12.45^°^ and all these porous materials are not crystalline polymers as illustrated in PXRD pattern (Figure 4).

### 2.2. Porosity

**Figure 5:**
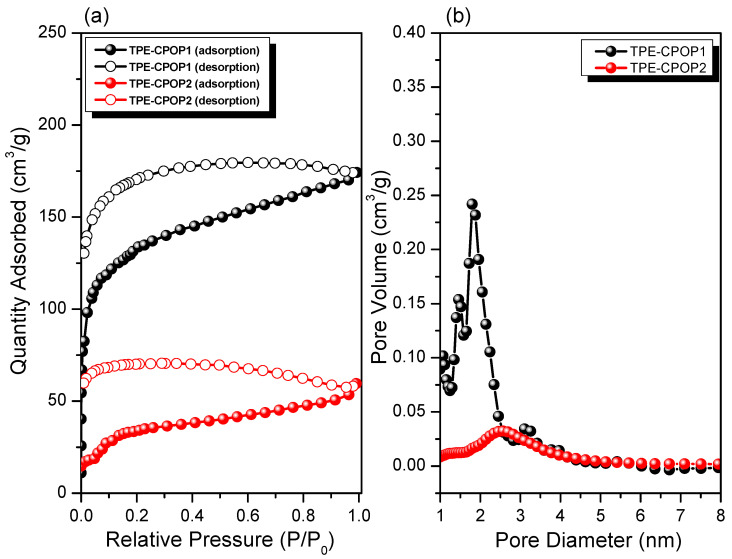
(**a**) N_2_ adsorption desorption pattern (filled cycles: adsorption; empty cycles: desorption) and (**b**) pore size diameter of TPE-CPOP1 and TPE-CPOP2.

The porosity properties like Brunauer–Emmett–Teller (BET) surface areas, total pore volume, and pore size diameter of TPE-CPOP1 and TPE-CPOP2 before carbonization and KOH activation process were characterized by N_2_ adsorption/desorption measurements at 77 K and 1 bar, as presented in Figure 5. Both the N_2_ adsorption isotherms of TPE-CPOP-1 and TPE-CPOP2 showed increase the N_2_ capture at low- and high-pressure P/P_0_ values indicating that both two materials curves could be classified as type I according to IUPAC classification (Figure 5a). In addition, the N_2_ adsorption isotherms of TPE-CPOP1 and TPE-CPOP2 at high-pressure values possesses a hysteresis loop, which indicates that the obtained polymer framework contains a mesoporous and microporous structure. Furthermore, the hysteresis loop for both materials does not close, which could be attributed to the flexibility network structure and the swelling of the frameworks during gas adsoprtion by elastic deformations [52]. The BET surface areas of TPE-CPOP1 and TPE-CPOP2 were found to be 489 and 146 m^2^ g^−1^, respectively, and their pore volumes were found to be 0.269 and 0.1 cm^3^ g^−1^ at P/P_0_ = 0.996, respectively. In addition, the pore size diameter was estimated by the nonlocal density functional theory (NLDFT) and the results showed that the pore size diameter was 1.49 and 1.82 nm, respectively, for TPE-CPOP1 and 2.57 nm for TPE-CPOP2 (Figure 5b). 

**Figure 6:**
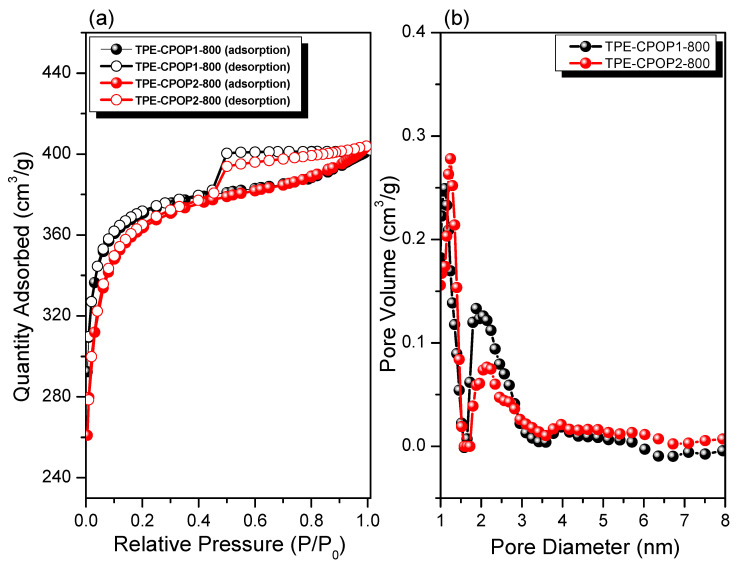
(**a**) N_2_ adsorption desorption pattern (filled cycles: adsorption; empty cycles: desorption) and (**b**) pore size diameter of TPE-CPOP1-800 and TPE-CPOP2-800.

As shown in Table 1, the BET surface area of TPE-CPOP2 lower than that of TPE-CPOP1, presumably because of the low attachment of triazine units into TPE moiety during the Friedel–Crafts reaction [65,66]. Figure 6 shows the N_2_ adsorption/desorption and pore size distribution of TPE-CPOP1-800 and TPE-CPOP2-800 to investigate their porous nature. As shown in Figure 6, both TPE-CPOP1-800 and TPE-CPOP2-800 exhibited a rapid N_2_ capture ability at low pressure and continued to increase for N_2_ adsorption at high-pressure regions, which indicated the presence of micropores and mesopores in the materials. Based on the IUPAC classification, the adsorption/desorption isotherm of TPE-CPOP1-800 and TPE-CPOP2-800 possesses type I and type IV. The values of BET surface area, total pore volume, and pore size diameter were 1177 m^2^ g^−1^, 0.48 cm^3^ g^−1^, and 1.04–2.99 nm, respectively, for TPE-CPOP1-800 and 1165 m^2^ g^−1^, 0.62 cm^3^ g^−1^, and 1.02–2.29 nm, respectively, for TPE-CPOP2-800. In addition, the lack of difference in surface area between TPE-CPOP1-800 and TPE-CPOP2-800 due to the KOH acts as an activation agent for TPE-CPOP1 and TPE-CPOP2 to produce carbon materials and enhance their porosity properties such as specific surface area, pore size, and total pore volume [67]. 

**Figure 7:**
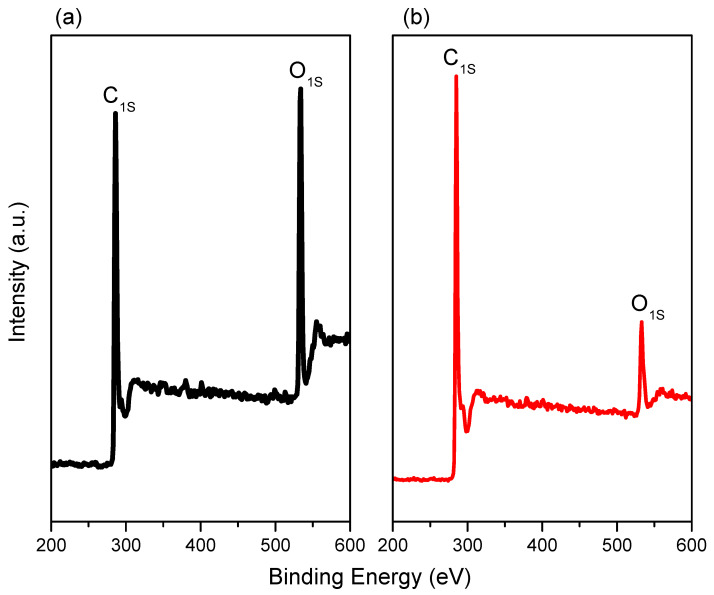
XPS profile of (**a**) TPE-CPOP1-800 and (**b**) TPE-CPOP2-800.

The surface morphologies of TPE-CPOP1-800 and TPE-CPOP2-800 showed ultra-micropours and these materials are amorphous based on TEM images as shown in Figure S5. The presence of the carbon and oxygen atoms in the surface of TPE-CPOP1-800 and TPE-CPOP2-800 was confirmed by XPS analysis (Figure 7). As displayed in Figure 7, the XPS survey spectra for both these materials possesses signals at 284 eV and 530 eV representing the carbon atoms of the aromatic rings and oxygen atoms in the microporous carbon materials. 

**Figure 8:**
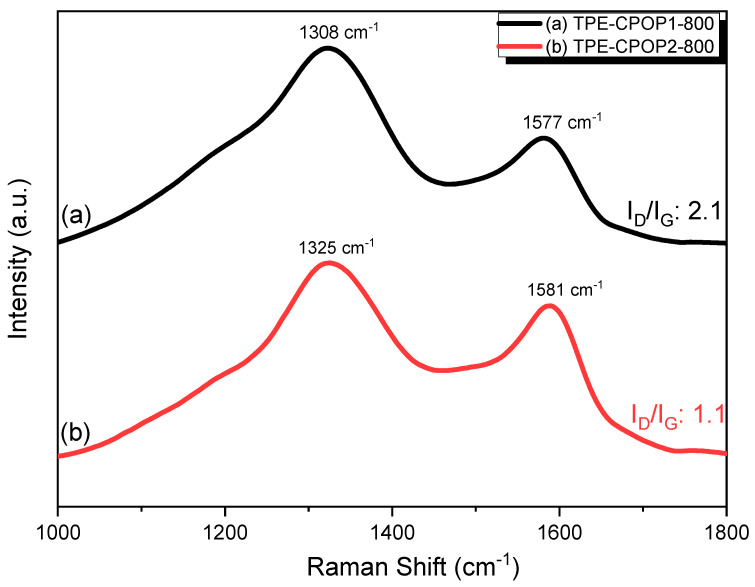
Raman profile of (**a**) TPE-CPOP1-800 and (**b**) TPE-CPOP2-800.

The Raman spectra (Figure 8) showed that TPE-CPOP1-800 exhibits a definite carbonized structure with two identical bands of D and G, which correspond to sp^3^ and sp^2^ carbons, respectively [68,69,70]. The D and G band positions were found at 1325.0 and 1580.9 cm^−1^, respectively. In addition, the I_D_/I_G_ ratio was found to be 1.5, which clearly describes that sp^3^ carbons are higher than sp^2^ carbons due to the structural functionalization. This indicated that the activation process did not destroy the chemical structure. In addition, upon activating TPE-CPOP2-800 the band position was shifted. In detail, D band was found at 1308.4 cm^−1^ and G band was at 1577.2 cm^−1^, which indicated a change in the Fermi energy level caused by the Lewis acid reaction [71,72]. In addition, the I_D_/I_G_ ratio reached 1.9 indicating further sp^3^ hybridizations was found upon the activation process possibly due to further functionalization occurred during the heating procedure. These results clearly indicated that the KOH activation did not destroy the graphitic structure and maintained the sp^2^ and sp^3^ hybridizations [11,73].

### 2.3. CO_2_ Uptake 

**Figure 9:**
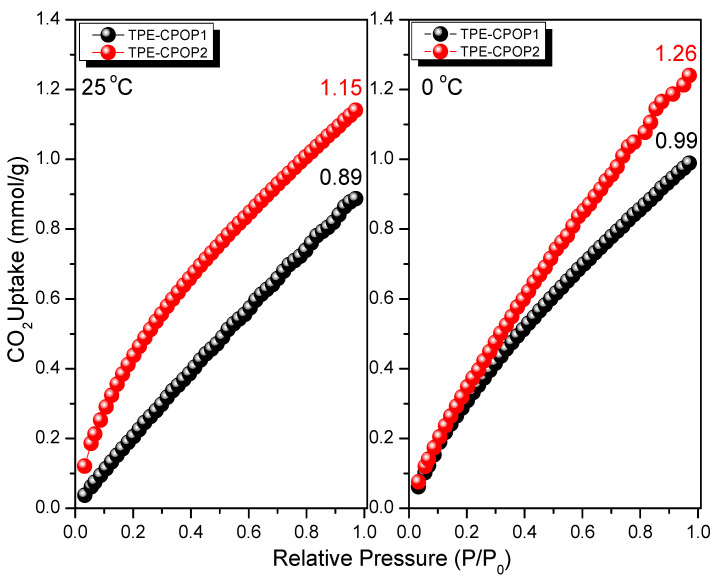
CO_2_ uptake of TPE-CPOP1 and TPE-CPOP2 at 25 °C (**a**) and 0 °C (**b**).

**Figure 10:**
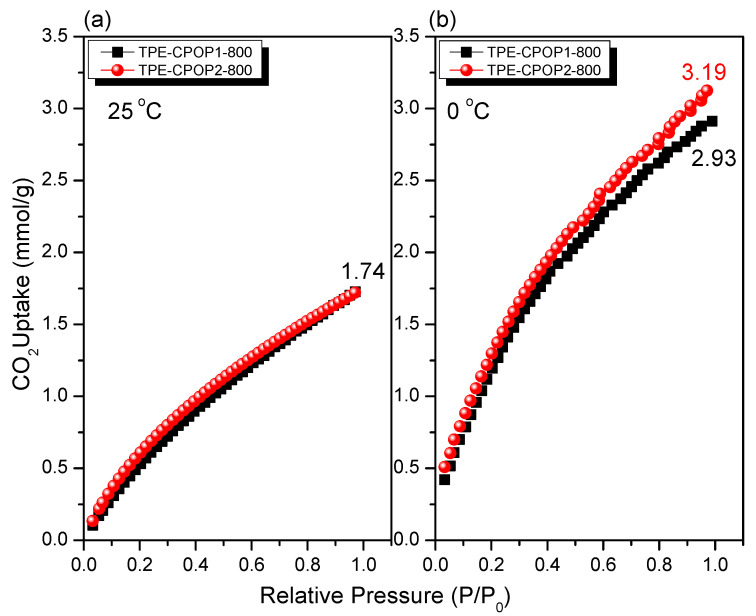
CO_2_ uptake of TPE-CPOP1-800 and TPE-CPOP2-800 at 25 °C (**a**) and 0 °C (**b**).

Based on BET results, our new materials TPE-CPOP1, TPE-CPOP2, and their resulting microporous carbon materials (TPE-CPOP1-800 and TPE-CPOP2-800), after carbonization and KOH activation process, feature high surface areas, large pore. volumes, and meso and microporous structures. Therefore, we expected that all these materials could be applied as candidates for gas capture and energy storage. The CO_2_ uptake performance of TPE-CPOP1, TPE-CPOP2, TPE-CPOP1-800, and TPE-CPOP2-800 were determined by CO_2_ isotherm measurements at 298 and 273 K, respectively (Figure 9 and Figure 10). The results revealed that the values of the CO_2_ adsorption capacity were found to be 0.89 and 1.15 mmol g^−1^ at 298 K for TPE-CPOP1 and TPE-CPOP2, respectively (Figure 9a). On the contrary, the adsoprtion capacity of CO_2_ at 273 K reached 0.99 and 1.26 mmol g^−1^ for TPE-CPOP1 and TPE-CPOP2, respectively (Figure 9b). We supposed the TPE-CPOP2 had higher and excellent CO_2_ uptake performance compared to TPE-CPOP1 due to the presence of triazine units in the TPE-CPOP2 framework, which facilitate the dipole–quadrupole interactions with the CO_2_ molecules. As previously reported, the improvement of CO_2_ uptake performance of porous polymers can be achieved by carbonization and KOH activation process at elevated temperatures [74]. Thus, we did the calcination and KOH activation process for TPE-CPOP1 and TPE-CPOP2 at 800 °C for 8 h under a N_2_ atmosphere to produce microporous carbon materials with high specific BET surface areas and pore size diameters. Interestingly, the values of CO_2_ uptake were 1.74 and 1.72 mmol g^−1^ at 298 K for TPE-CPOP1-800 and TPE-CPOP2-800, respectively (Figure 10a). At 273 K, the CO_2_ adsorption capacity reached 3.19 and 2.93 mmol g^−1^ for the TPE-CPOP1-800 and TPE-CPOP2-800, respectively (Figure 10b). We revealed that both TPE-CPOP1-800 and TPE-CPOP2-800 showed higher CO_2_ adsorption capacity that than that of the Th850 (2.4 mmol at 298 K) [74], which can be attributed to their high specific surface areas (up to 1100 m^2^ g^−1^) and total pore volume (up to 0.48 cm^3^ g^−1^).

### 2.4. Electrochemical Performance

**Figure 11:**
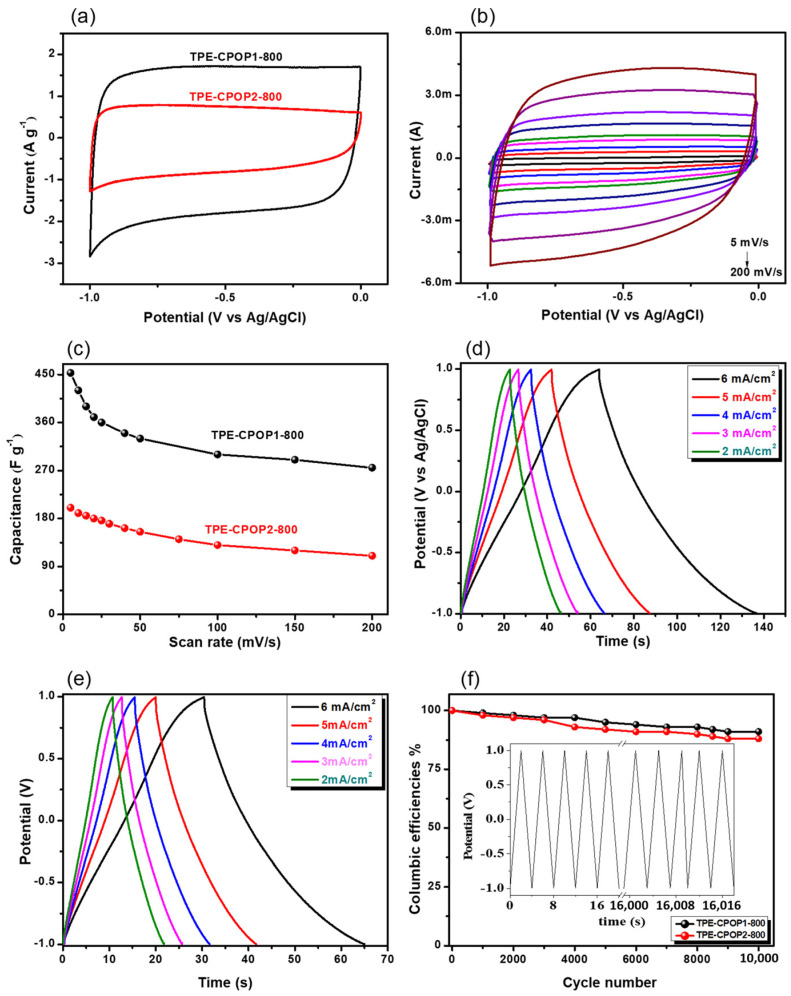
(**a**) Comparison of CV performance between TPE-CPOP1-800 and TPE-CPOP2-800 at 50 mV/s. (**b**) CV performance of TPE-CPOP1-800 at various scan rates (5~200 mV s^−1^). (**c**) Capacitance performance of TPE-CPOP1-800 and TPE-CPOP2-800 at various scan rates. (**d**) Galvanic charge/discharge (GCD) performance of TPE-CPOP1-800. (**e**) GCD performance of TPE-CPOP2-800. (**f**) Columbic efficiencies of TPE-CPOP1-800 and TPE-CPOP2-800 at 20 A g^−1^ for 10,000 cycles. The inset figure represents the first and last few cycles of GCD behavior at 20 A g^−1^.

The electrochemical characteristics of the TPE-CPOP1-800 and TPE-CPOP2-800 were investigated using cyclic voltammetry (CV), charge/discharge (CD) and cycling stability. The CV was tested over the potential range between –1.0 and 0.0 V at various scan rates of 5 mV/s up to 200 mV/s (Figure 11a). The CV shape of the TPE-CPOP1-800 and TPE-CPOP2-800 showed a perfect electric double layer behavior all over the potential range and at all investigated scan rates. It is important to notice the difference between the two cases of KOH activation_;_ as it can be clearly seen in (Figure 11b), since the TPE-CPOP1-800 showed an almost double area of the CV loop at all investigated scan rates compared to TPE-CPOP2-800. This is clear evidence of the importance of a synergistic effect upon the activation processes in enhancing the electrochemical performances without defecting the main EDLC of the carbon structure [73,75]. The activation process provided an efficient increase in the surface area of the obtained porous carbon materials. The main difference between both porous carbon materials is the existence of the triazine moiety in the TPE-CPOP2 that is expected to have a strong steric hindrance that reduced the effect of KOH activation reaction to reach the inner carbon structure. Therefore, the obtained surface area of TPE-CPOP2 upon KOH activation was less than TPE-CPOP1-800. Moreover, upon plotting the capacitance versus scan rates, the achieved capacitance value for the TPE-CPOP1-800 reached 453 F g^−1^, which was 200 F g^−1^ for TPE-CPOP2-800 at the same scan rate of 5 mV/s (Figure 11c). It is believed that the well-designed porous structure in TPE-CPOP1-800 was yielded by the efficient electron transfer between KOH and TPE-CPOP1 that could promote the surface area of the porous carbon structure and provide better electron transfer efficiencies leading to an outstanding EDLC performance than other reported porous carbons including e.g., porous carbons derived from poly(caprolactone–b–ethylene oxide–b–caprolactone) triblock copolymer that reached a capacitance of 90 F g^−1^ at 5 mV s^−1^ [76]. In addition, both TPE-CPOP1-800 and TPE-CPOP2-800 exhibit dual pore features. However, TPE-CPOP1-800 has higher pore size (2.99 nm) than that of TPE-CPOP2-800 (2.29 nm) and this is leading to the rapid transfer of electrolyte ions at the interface between the electrolyte and the electrode [11,77,78]. In addition, this capacitance value is still higher than other reported results of porous carbon derived from natural resources of jackfruit seed and sorghum biomass-derived porous carbons, which achieved 292.2 F g^−1^ and 240 F g^−1^, respectively, at 5 mV s^−1^ [79,80]. Activation of Lapsi seed yielded porous carbon with high surface area of 1316.7 m^2^ g^−1^ with a capacitance of 317.5 F g^−1^ at 5 mV s^−1^ [81]. It is interesting to compare the effect of different Lewis acids e.g., FeCl_3_ in graphite intercalated compounds when reacted with dodcecyl amine and heated at higher temperatures of 900 °C and 2000 °C without activation to accomplish a surface area of 17 and 53 m^2^ g^−1^ and a capacitance of 42 and 90 F g^−1^, respectively [82,83]. Therefore, it is important to note the effect of KOH as activating agent here to provide such an enormous enhancement in the obtained surface area and electrochemical capacitance performances that would have been impossible without KOH activation. As presented in Table S1, The TPE-CPOP1-800 displayed the highest capacitance values compared with other porous carbon materials. In addition, the charge/discharge behavior was investigated at a wider potential range of (−1.0 to 1.0 V) to obtain a clear overview of the full potential range. The charge/discharge showed a symmetric behavior with no obvious IR drop at all investigated current densities. This indicated that there were no obvious defects in the structure after the activation process occurred [84]. Moreover, similar to the CV results, the charge/discharge behavior was compared for both TPE-CPOP1-800 and TPE-CPOP2-800 after the activation process, it was found that the activation process for TPE-CPOP1-800 yielded a double efficiency compared to the activated TPE-CPOP2-800 carbons at all investigated current densities (Figure 11d,e). These results also confirmed the CV results that triazine moiety has decreased the efficiencies of the KOH activation process in the electrochemical performance. Moreover, the columbic efficiencies were also compared for both conditions. It was found that the TPE-CPOP1-800 showed efficient stability of 96% as average stability upon cycling for 10,000 cycles at 20 A g^−1^, however the TPE-CPOP2-800 conditions only showed 93% of the average stability at the same current densities (Figure 11e,f). These results are supporting the previous results found from both CV and charge/discharge behavior. It also showed the efficient stabilities of both TPE-derived carbons after long cycles capacities and higher current densities. The calculated energy density vs. scan rates showed efficient energy densities values for TPE-CPOP1-800 at all scan rates as displayed in Figure S6. The energy density achieved 63 Wh Kg^−1^ at 5 mV s^−1^, which is mainly due to the efficient electrical double layer capacitor (EDLC) capacitive performance within the wide potential window. This value is considered much higher than other related graphene composites that achieved a maximum energy density of 46 Wh Kg^−1^ at 5 mV s^−1^ without activation [82,85]. Therefore, it is believed that the KOH activation has a unique characteristic to enrich the carbon materials with excellent electrochemical properties for high capacitive and high energy densities performances. We believe that this method will open the door for further investigations of activation procedures for various carbon-derived polymers for efficient energy storage applications. 

## 3. Materials and Methods

### 3.1. General Information

Benzophenone (99%), potassium carbonate (K_2_CO_3_, 99.9%), titanium tetrachloride (TiCl_4_, 99.9%), zinc (Zn, 98%), and 1,2-dichloroethane (DCE, 99.8%) were ordered from Alfa Aesar. Anhydrous magnesium sulfate (MgSO_4_, 99.5%), ethylacetate (EA), tetrahydrofuran (THF), acetone, methanol (CH_3_OH), and dichloromethane (DCM) were purchased from Showa (Tokyo, Japan).

### 3.2. Synthesis

TPE: Tetraphenylethene (TPE) monomer was successfully synthesized according to our previous report [47,50]. FTIR (KBr, cm^−1^, Figure S1): 3047 (aromatic C–H stretching), 1602 (C=C stretching). ^1^H-NMR (500 MHz, δ, ppm, CDCl_3_, Figure S2): 7.05–7.15 (m, 20H). ^13^C-NMR (125 MHz, δ, ppm, CDCl_3_, Figure S3): 140.7; 141.0; 131.3; 127.7; 126.4.

TPE-CPOP1: A solution tetraphenylethene (0.50 g, 1.51 mmol),) and AlCl_3_ (0.20 g, 1.51 mmol) in dry 1,2-dichloroethane (20 mL). The mixture was stirred at room temperature for 0.5 h and then refluxed at 60 °C for 24 h. After cooling to 25 °C, the brown solid was filtered and washed three times with chloroform, dichloromethane, methanol, THF, and acetone to remove the unreacted TPE and AlCl_3_ and dried under vacuum at 60 °C to give TPE-CPOP1 as a brown powder (0.45 g, 90%).

TPE-CPOP2: A solution of tetraphenylethene (0.50 g, 1.51 mmol), cyanuric chloride (0.37 g, 2 mmol), and AlCl_3_ (0.20 g, 1.51 mmol) in dry 1,2-dichloroethane (20 mL) was stirred at room temperature for 0.5 h and then heated at 60 °C for 24 h. After cooling at room temperature, the solid was filtered and washed three times with THF, methanol, chloroform, and acetone to remove the unreacted monomer and AlCl_3_ and dried under vacuum at 60 °C to obtain chloroform, dichloromethane, methanol, THF, and acetone to remove the unreacted TPE and AlCl_3_ and dried under vacuum at 60 °C to get TPE-CPOP2 as brown solid (0.4 g, 80%).

Preparation of TPE-CPOP1-800 and TPE-CPOP2-800: 0.4 g of TPE-CPOP1 or TPE-CPOP2 and 0.4 g of KOH were mixed in 3 mL of water and the mixture was stirred for 5 h at room temperature. After that, the water solution was removed from the mixture at 120 °C for 24 h. Then, the dried sample powder was calcinated in the furnace at 800 °C for 8 h under a N_2_ atmosphere (a heating rate of 5 °C min^−1^). After cooling to RT, the black solid was washed with 2 N HCl, water, THF, methanol, and acetone, respectively, to give TPE-CPOP1-800 (0.25 g, 63%) and TPE-CPOP2-800 (0.27 g, 68%).

## 4. Conclusions

In summary, two kinds of HCPs (TPE-CPOP1 and TPE-CPOP2) were successfully prepared through the simple and friendly Friedel−Crafts polymerization of tetraphenylethene with or without cyanuric chloride in the presence of AlCl_3_ as a catalyst. Their chemical structures were confirmed by FTIR and NMR analyses. Interestingly, the obtained TPE-CPOP1-800 porous materials after the carbonization and KOH activation displayed good thermal stability, high surface area, excellent CO_2_ adsorption capacity (1.74 mmol g^−1^ at 298 K and 3.19 mmol g^−1^ at 273 K), a high specific capacitance of 453 F g^−1^ at 5 mV s^−1^, and efficient stability of 96% as average stability upon cycling for 10,000 cycles.

## Figures and Tables

**Table 1:** Thermal stability and porosity properties of TPE, TPE-CPOP1, TPE-CPOP2, TPE-CPOP1-800, and TPE-CPOP2-800.

Sample	T_d5_ (°C)	T_d10_ (°C)	Char Yield (wt%)	Surface Area (m^2^ /g)	Pore Size (nm)
TPE-CPOP1	412	519	69	489	1.49, 1.82
TPE-CPOP2	248	365	67	146	2.57
TPE-CPOP1-800	356	464	70	1177	1.04, 2.99
TPE-CPOP2-800	375	500	72	1165	1.02, 2.29

## Data Availability

The data presented in this study are available in the article and supplementary material.

## Supplementary Materials

The following are available online, Figure S1. FT-IR spectrum of TPE. Figure S2. ^1^H NMR spectrum of TPE. Figure S3. ^13^C NMR spectrum of TPE. Figure S4. XPS profile of (a) TPE-CPOP1and (b) TPE-CPOP2. Figure S5. TEM images of (a) TPE-CPOP1-800 and (b) TPE-CPOP2-800. Figure S6. Energy density vs. scan rate for TPE-COP1-800 and TPE-COP2-800. Table S1. comparison list of other activated carbon materials.

## Sample Availability

Samples of the compounds are not available from the authors.
