# Supplementary material for: Meso/Microporous Carbons from Conjugated Hyper-Crosslinked Polymers Based on Tetraphenylethene for High-Performance CO2 Capture and Supercapacitor
Source: Molecules. 2021 Jan 31;26(3):738. doi: 10.3390/molecules26030738 (PMC7866987; doi:10.3390/molecules26030738)
Supplement: Supplementary file 1 [file molecules-26-00738-s001.pdf]

Meso/Microporous Carbons from Conjugated Hyper-cross-linked Polymers based on Tetraphenylethene for High-Performance CO_2 Capture and Supercapacitor

Mohamed Gamal Mohamed [1,2], Mahmoud M.M. Ahmed [1,3], Wei-Ting Du [1] and Shiao-Wei Kuo [1,4,*]

[1] Department of Materials and Optoelectronic Science, Center of Crystal Research, National Sun Yat-Sen University, Kaohsiung 80424, Taiwan; mgamal.eldin34@gmail.com (M.G.M.); mahmoud.ahmed@mail.ntust.edu.tw (M.M.M.A.); justguita@gmail.com (W.-T.D.)
[2] Chemistry Department, Faculty of Science, Assiut University, Assiut 71516, Egypt
[3] Chemistry Department, Chung Yuan Christian University, Taoyuan 320, Taiwan
[4] Department of Medicinal and Applied Chemistry, Kaohsiung Medical University, Kaohsiung 807, Taiwan
* Correspondence: kuosw@faculty.nsysu.edu.tw

Characterizations

FTIR spectra were recorded using a Bruker Tensor 27 FTIR spectrophotometer and the conventional KBr disk method; 32 scans were collected at a spectral resolution of 4 cm^{-1}. The films used in this study were sufficiently thin to obey the Beer–Lambert law. Wide-Angle X-ray diffraction (WAXD) pattern was obscured from the wiggler beamline BL17A1 of the National Synchrotron Radiation Research Center (NSRRC), Taiwan. A triangular bent Si (111) single crystal was used to obtain a monochromated beam with a wavelength (λ) of 1.33 Å. Cross-polarization with MAS (CP/MAS) was used to acquire ^{13}C NMR spectral data at 75.5 MHz. The CP contact time was 2 ms; $_1H$ decoupling was applied during data acquisition. The decoupling frequency corresponded to 32 kHz. The MAS sample spinning rate was 10 kHz. Transmission electron microscope (TEM) images were obtained with a JEOL JEM-2010 instrument operated at 200 kV. Field emission scanning electron microscopy (FE-SEM) was conducted using a JEOL JSM7610F scanning electron microscope. Samples were treated via Pt sputtering for 100 s before observation. BET surface area and porosimetry measurements of the prepared samples (ca. 40–100 mg) were performed using a BEL. Nitrogen isotherms were generated through incremental exposure to ultrahigh-purity N_2 (up to ca. 1 atm) in a liquid nitrogen (77 K) bath. Surface parameters were determined using BET adsorption models in the instrument's software. TGA was performed using a TA Q-50 analyzer under a flow of N_2 atmosphere. The samples were sealed in a Pt cell and heated from 40 to 800 °C at a heating rate of 20 °C min^{-1} under a flow of N_2 atmosphere at a flow rate of 60 mL min^{-1}.UV–Vis spectra were recorded at 25 °C using a Jasco V-570 spectrometer, with deionized water as the solvent. The Raman spectra were investigated using Horiba Jobin-Yvon HR800 Raman Spectrometer with 633 nm laser, 10 sec accumulated scans repeated for 20 times, and 50x magnification lens. The electrochemical performances were performed using Zahner Zennium E electrochemical workstation with three electrodes configurations using Ag/AgCl as a reference electrode and 1.0 M KCl aqueous electrolyte medium. The working electrode was an ITO substrate coated by the slurry of the tested material [1]. The cyclic voltammetry (CV) was tested over the range between −1.0 and 0.0 V and the scan rates were investigated from 5 mV s^{-1} up to 200 mV s^{-1}. The galvanic charge/discharge (GCD) performance was performed at the same instrumental setup within the range of −1.0 to 1.0 V at various current densities. The columbic efficiencies were tested at 20 A g^{-1} for 10,000 cycles.

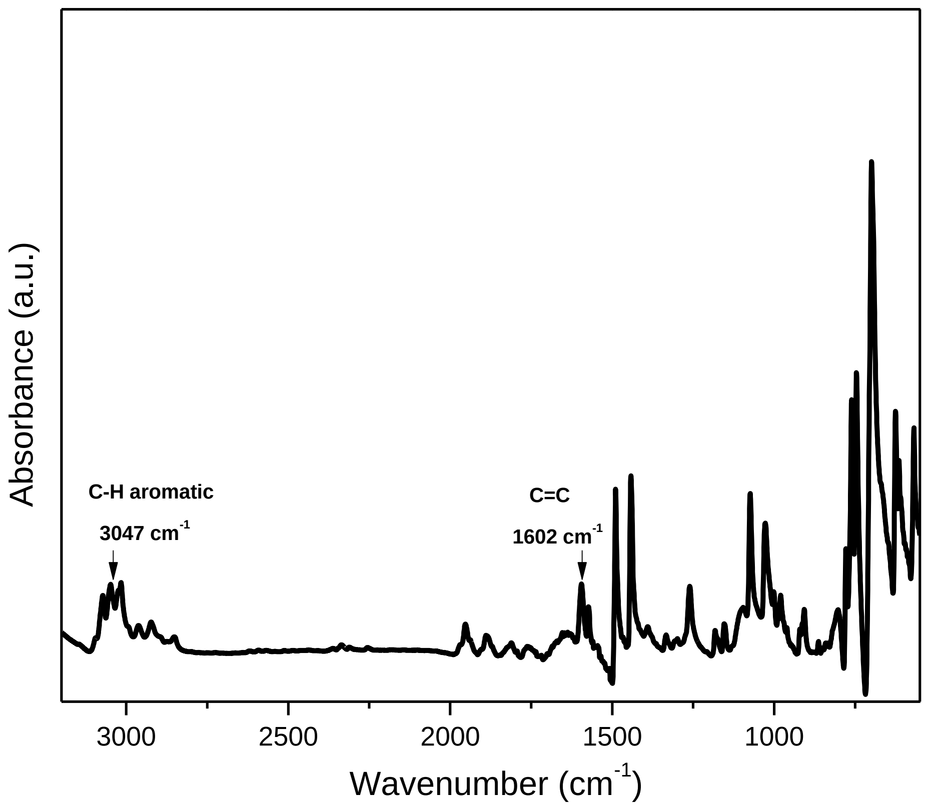

Figure S1. FT-IR spectrum of TPE.

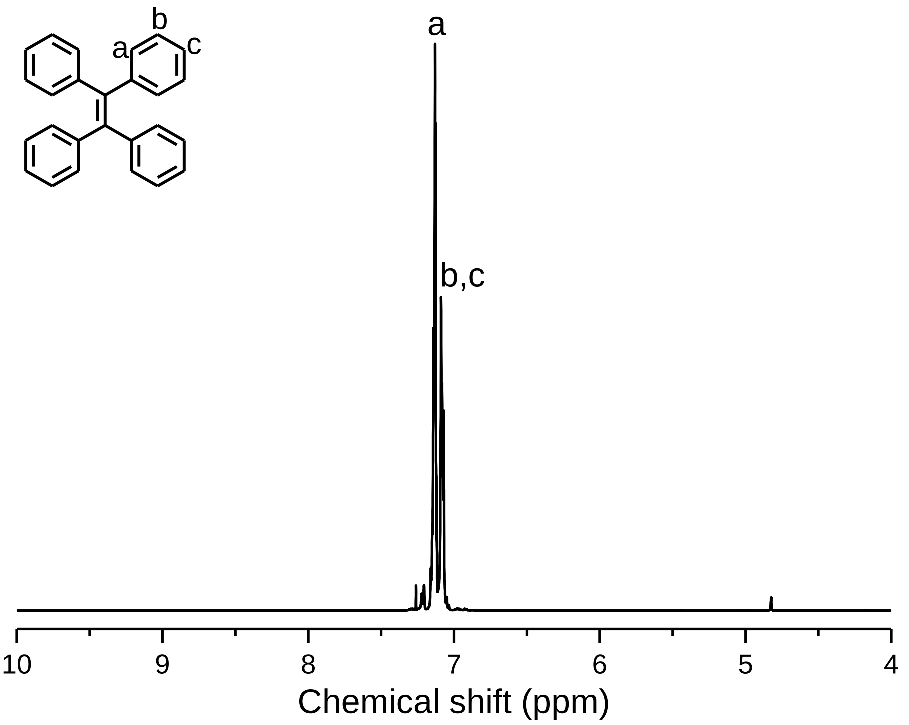

Figure S2. ^{1}H NMR spectrum of TPE.

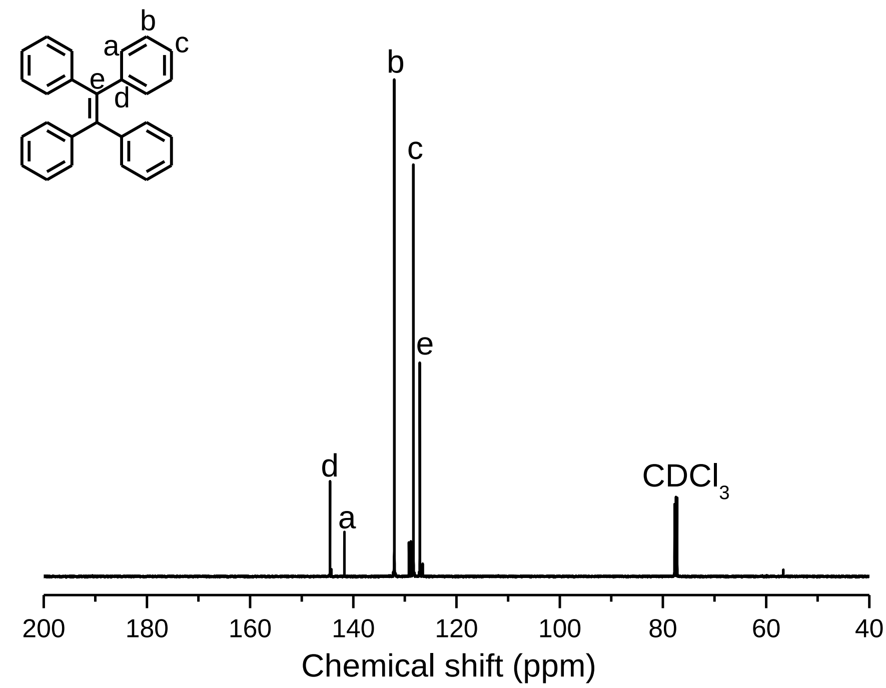

Figure S3. ^{13}C NMR spectrum of TPE.

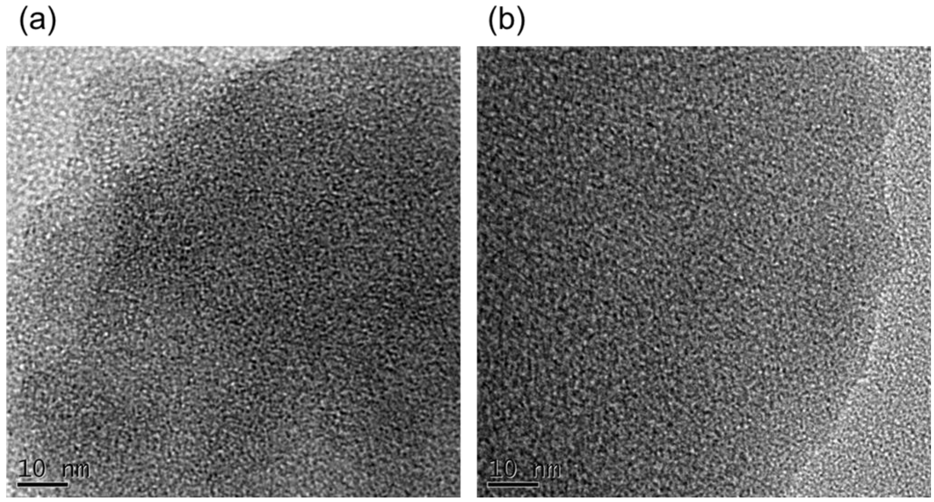

Figure S4. TEM images of (**a**) TPE-CPOP1-800 and (**b**) TPE-CPOP2-800.

Table S1. Comparison list of other activated carbon materials.

Material	Surface Area m^2g^{-1}	Capacitance	Ref
Conversion of fullerene C70 cubes (MCFC) into mesoporous carbon	642.6	286 F g^{-1}/5 mV s^{-1}	[1]
Lotus Seed Derived Nanoporous Carbon	1316.7	317.5 F g^{-1}/5 mV s^{-1}	[2]
Sorghum biomass-derived porous carbon	1674	228 F g^{-1}/5 mV s^{-1}	[3]
Phenolic formaldehyde resin with sodium citrate	1256	261 F g^{-1}/0.05 A g^{-1}	[4]
cross-linked lignin gel impregnated with a surfactant	1148	100 Fg^{-1}/5mVs^{-1}	[5]
Asphaltene-Based Porous Carbon	2168	135.4 Fg^{-1}/1 Ag^{-1}	[6]
Pillared graphene	330	200 Fg^{-1}	[7]
Heating at 1000 °C in Ar atmosphere for graphite intercalated compound (GIC)	-	216 Fcm^{-1}	[8]

Heating exfoliated GIC at 2000 °C	50	90 F g^{-1}/5mVs^{-1}	[9]
Pyrolysis of pistachio nutshell biomass	1900	45 F g^{-1}/ 1 mVs^{-1}	[10]
Hollow carbon-MoS2-carbon nanoplates	543	178 Fg^{-1}/1.0 A g^{-1}	[11]
activated carbon and carbon fiber yarns	1600	45.2 mF cm^{-1}/2 mV s^{-1}	[12]
Mesoporous Nitrogen-Doped Hollow Carbon Nanoplates with Uniform Hexagonal Morphologies	460	95 Fg^{-1}/1.0 A g^{-1}	[13]
Carbon nanotube composite with activated carbon	-	60.75 F cm^{-3}	[14]
Lignin-Based and Cellulose Hydrogels	713	292 F g^{-1}/0.5 A g^{-1}	[15]
Nanoholes on Single-Walled Carbon Nanohorn	1020	8.6 μF cm^{-2}	[16]
tannic acid (TA) and carbon nanotubes (CNTs)	-	147.4 Fg^{-1}/ 0.5 A g^{-1}	[17]
Carbon Composite and Replica Obtained from Hybrid Layered Double Hydroxide Active Container	1535	92.6 μF cm^{-2}	[18]
Carbons derived from Peach Gum	95	199 F g^{-1}/0.2 A g^{-1}	[19]
Mesoporous graphitic carbon microtubes derived from fullerene C_{70} through heating at 2000 °C	609	212.2 F g^{-1}/5 mV s^{-1}	[20]
TPE-CPOP1-800	1177	453 F g^{-1}/5 mV s^{-1}	This work
TPE-CPOP2-800	1165	200 F g^{-1}/5 mV s^{-1}	This work

References

1. Bairi, P.; Maji, S.; Hill,J.P.; Kim, J.H.; Ariga, K.; Shrestha, L.K. Mesoporous carbon cubes derived from fullerene crystals as a high rate performance electrode material for supercapacitors, *J. Mater. Chem. A* **2019**, *7*, 12654–12660.
2. Shrestha, R.L.; Chaudhary, R.; Shrestha, T.; Tamrakar, B.M.; Shrestha, R.G.; Maji, S.; Hill, J.P.; Ariga, K.; Shrestha, L.K. Nanoarchitectonics of Lotus Seed Derived Nanoporous Carbon Materials for Supercapacitor Applications. *Materials* **2020**, *13*, 5434.
3. Kim, M.; Lim, H.; Xu, X.; Hossain, M.S.A.; Na, J.; Awaludin, N.N.; Shah, J.; Shrestha, L.K.; Ariga, K.; Nanjundan, A.K.; Martin, D.J.; Shapter, J.G.; Yamauchi, Y. Sorghum biomass-derived porous carbon electrodes for capacitive deionization and energy storage. *Micropor. Mesopor. Mat*. **2021**, *312*, 110757.
4. Zhang, J.; Zhang, W.; Han, M.; Pang, J. One pot synthesis of nitrogen-doped hierarchical porous carbon derived from phenolic formaldehyde resin with sodium citrate as activation agent for supercapacitors. J. *Mater. Sci.: Mater. Electron*. **2018**, *29*, 4639–4648.
5. Saha, D.; Li, Y.; Bi, Z.; Chen, J.; Keum, J.K.; Hensley, D.K.; Grappe, H.A.; Meyer, H.M.; S. Dai, S.; Paranthaman, M.P.; Naskar, A.K. Studies on Supercapacitor Electrode Material from Activated Lignin-Derived Mesoporous Carbon. *Langmuir* **2014**, *30*, 900–910.
6. Qin, F.; Tian, X.; Guo, Z.; Shen, W. Asphaltene-based porous carbon nanosheet as electrode for supercapacitor. *ACS Sustain. Chem. Eng*. **2018**, *6*, 15708–15719.
7. Banda, H.; Périé, S.; Daffos, B.; Taberna, P.L.; Dubois, L.; Crosnier,O.; Simon, P.; Lee, D.; De Paëpe, G.; Duclairoir, F. Sparsely Pillared Graphene Materials for High-Performance Supercapacitors: Improving Ion Transport and Storage Capacity. *ACS Nano* **2019**, *13*, 1443–1453.
8. Wee, B.H.; Wu, T.F.; Hong, J.D. Facile and scalable synthesis method for high-quality few-layer graphene through solution-based exfoliation of graphite. *ACS Appl. Mater. Interfaces* **2017**, *9*, 4548–4557.
9. Ahmed, M.M.M.; Imae, T.; Hill, J.P.; Yamauchi, Y.; Ariga, K.; Shrestha, L.K. Defect-free exfoliation of graphene at ultra-high temperature. *Colloids Surf. A Physicochem. Eng. Asp*. **2018**, *538*, 127–132.
10. Goldfarb, J.L.; Dou, G.; Salari, M.; Grinstaff, M.W. Biomass-Based Fuels and Activated Carbon Electrode Materials: An Integrated Approach to Green Energy Systems. *ACS Sustain. Chem. Eng*. **2017**, *5*, 3046–3054.
11. Quan, T.; Goubard-Bretesché, N.; Härk, E.; Kochovski, Z.; Mei, S.; Pinna, N.; Ballauff, M.; Lu, Y. Highly Dispersible Hexagonal Carbon–MoS_2–Carbon Nanoplates with Hollow Sandwich Structures for Supercapacitors. *Chem. Eur. J*. **2019**, *25*, 4757–4766.
12. Zhai, S.; Jiang, W.; Wei, L.; Karahan, H.E.; Yuan, Y.; Ng, A.K.; Chen, Y. All-carbon solid-state yarn supercapacitors from activated carbon and carbon fibers for smart textiles. *Mater. Horiz*. **2015**, *2*, 598–605.
13. Cao, J.; Jafta, C.J.; Gong,J.; Ran, Q.; Lin, X.; Félix, R.; Wilks, R.G.; Bär, M.; Yuan, J.; Ballauff, M.; Lu, Y. Synthesis of Dispersible Mesoporous Nitrogen-Doped Hollow Carbon Nanoplates with Uniform Hexagonal Morphologies for Supercapacitors. *ACS Appl. Mater. Interfaces* **2016**, *8*, 29628–29636.
14. Park, H.; Ambade, R.B.; Noh, S.H.; Eom, W.; Koh, K.H.; Ambade, S.B.; Lee, W.J.; Kim, S.H.; Han, T.H. Porous Graphene-Carbon Nanotube Scaffolds for Fiber Supercapacitors. *ACS Appl. Mater. Interfaces* **2019**, *11*, 9011–9022.
15. Peng, Z.; Zou, Y.; Xu, S.; Zhong, W.; Yang, W. High-Performance Biomass-Based Flexible Solid-State Supercapacitor Constructed of Pressure-Sensitive Lignin-Based and Cellulose Hydrogels. *ACS Appl. Mater. Interfaces* **2018**, *10*, 22190–22200.

16. Yang, C.M.; Kim, Y.J.; Miyawaki, J.; Kim, Y.A.; Yudasaka, M.; Iijima, S.; Kaneko, K. Effect of the Size and Position of Ion-Accessible Nanoholes on the Specific Capacitance of Single-Walled Carbon Nanohorns for Supercapacitor Applications. *J. Phys. Chem. C* **2015**, *119*, 2935–2940.
17. Oh, J.Y.; Jung, Y.; Cho, Y.S.; Choi, J.; Youk, J.H.; Fechler, N.; Yang,S.J.; Park, C.R. Metal–Phenolic Carbon Nanocomposites for Robust and Flexible Energy-Storage Devices. *ChemSusChem* **2017**, *10*, 1675–1682.
18. Stimpfling, T.; Leroux, F. Supercapacitor-Type Behavior of Carbon Composite and Replica Obtained from Hybrid Layered Double Hydroxide Active Container. *Chem. Mater*. **2010**, *22*, 974–987.
19. Lin, Y.; Chen, Z.; Yu, C.; Zhong, W. Heteroatom-Doped Sheet-Like and Hierarchical Porous Carbon Based on Natural Biomass Small Molecule Peach Gum for High-Performance Supercapacitors. *ACS Sustain. Chem. Eng*. **2019**, *7*, 3389–3403.
20. Bairi, P.; Shrestha, R.G.; Hill, J.P.; Nishimura, T.; Ariga, K.; Shrestha, L.K. Mesoporous graphitic carbon microtubes derived from fullerene C70 tubes as a high performance electrode material for advanced supercapacitors. *J. Mater. Chem. A* **2016**, *4*, 13899–13906.
